# Travel Behavior Change in Older Travelers: Understanding Critical Reactions to Incidents Encountered in Public Transport

**DOI:** 10.3390/ijerph121114741

**Published:** 2015-11-18

**Authors:** Catherine Sundling

**Affiliations:** Department of Psychology, Stockholm University, Gösta Ekman Laboratory, Stockholm SE-106 91, Sweden; E-Mail: catherine.sundling@psychology.su.se; Tel.: +46-8-164-601; Fax: +46-8-159-342

**Keywords:** older persons, critical incidents, critical reactions, travel behavior change

## Abstract

Accessibility of travel may be better understood if psychological factors underlying change in travel behavior are known. This paper examines older (65+) travelers’ motives for changing their travel behavior. These changes are grounded in critical incidents earlier encountered in public-transport travel. A scientific framework is developed based on cognitive and behavioral theory. In 29 individual interviews, travelers’ critical reactions (*i.e.*, cognitive, emotional, and/or behavioral) to 77 critical incidents were examined. By applying critical incident technique (CIT), five reaction themes were identified that had generated travel-behavior change: firm restrictions, unpredictability, unfair treatment, complicated trips, and earlier adverse experiences. To improve older travelers’ access to public transport, key findings were: (a) service must be designed so as to strengthen the feeling of being in control throughout the journey; (b) extended personal service would increase predictability in the travel chain and decrease travel complexity; consequently, (c) when designing new services and making effective accessibility interventions, policy makers should consider and utilize underlying psychological factors that could direct traveler behavior.

## 1. Introduction

Public transport should be accessible to all, though it is not obvious how this should be achieved. The proportion of journeys made by older people is expected to increase in many developed countries because of the increasing proportion of older people [1,2]. For example, almost 25% of the Swedish population is expected to be more than 65 years old in 2060, *versus* 19% in 2011 [3]. Functional limitations become more common with age, and many older persons will have acquired more than one such limitation [4]. In a group of older respondents and those entitled to Special Transport Service (a taxi service for disabled), 75% considered participation in society to be restricted because of problems encountered on the way to and from the bus stop or when entering/exiting the bus [5]. Accessible public transport contributes to wellbeing because it provides meaningful social interactions, a sense of community belonging, and reduced loneliness [6]. However, matching policy interventions to user needs requires more research into what barriers and facilitators are encountered when traveling, how they are experienced, and how they will affect future travel decisions.

## 2. Research Problem

This paper explores the link between critical incidents (*i.e.*, barriers/facilitators) and travel behaviors, as regards the following questions:

1. How do critical reactions (*i.e.*, cognitions, emotions, and behavior) to critical incidents encountered in public transport contribute to the older traveler’s process of travel behavior change?
2. How should older travelers’ critical reactions be understood, theoretically, in a cognitive and behavioral framework grounded in person–environment interaction?

Older people constitute a highly heterogeneous group. For example, the “older old”, *i.e.*, those aged 75 years and above, are less satisfied with their opportunities to travel than are the “younger old”, *i.e.*, those aged 65–74 years [7]. Moreover, those without a driver’s license, those living in rural areas, and women have all been found to have unfulfilled travel needs [8].

## 3. Theoretical Framework

**Figure 1:**
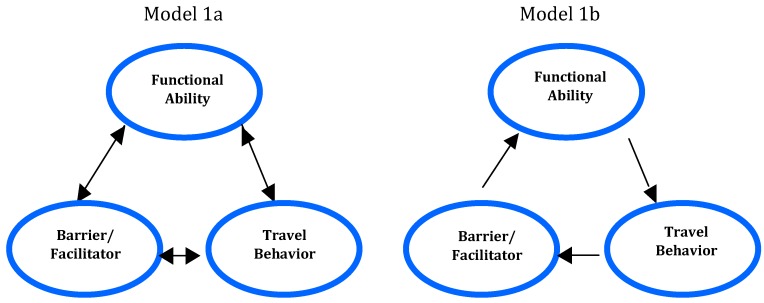
Model 1a is a general reciprocal model of accessibility and Model 1b is a unidirectional model of travel-behavior change.

Many possible determinants of travel behavior (in this paper referring to observable actions of travelers, *cf.* [9]) are discussed in the scientific literature. Some of these concern age and gender as well as socioeconomic and environmental factors [10]. Travel frequency has been found to decrease with age, especially at age 75 and above [11], although walking is an exception [10]. In the United States, Boschmann and Brady [12] found that, with increasing age (above 60+), travelers make fewer and shorter trips, women make fewer and shorter trips than men, and persons with disabilities make the fewest trips compared with all other persons. Moreover, the distance traveled was also found to increase with household size. The main focus in the present paper is on the *psychological* factors that determine individuals’ travel behavior, viewed in a cognitive and behavioral framework of *person–environment interaction* (e.g., [13]). Socioeconomic factors are beyond the scope of this paper and the environment will primarily be viewed from the traveler’s perspective. In the present research, the starting point is a reciprocal model of accessibility, in which barriers/facilitators, functional ability (including functional limitations), and travel behavior all affect each other (see Figure 1, Model 1a) [4,14]. This model is inspired by Bandura’s social-cognitive theory [15] and may be compared to the ecological model of Lawton and Nahemow (see further [16]), according to which a balance may be achieved by changing the individual’s capacity, the environmental demands, or both. In a similar study by Jensen, Iwarsson, and Ståhl [16], critical incidents were collected [17]. Accessibility was found to depend on a person’s functional limitations and on physical–environment factors. In this research, I focus specifically on how travel behavior changes, so a unidirectional model of *travel behavior change* is used (Figure 1, Model 1b). This model suggests that barriers/facilitators, experienced as *critical incidents*, may affect the traveler’s *perceived functional ability* and may result in changed travel behavior.

### 3.1. The Process of Travel-Behavior Change

**Figure 2:**
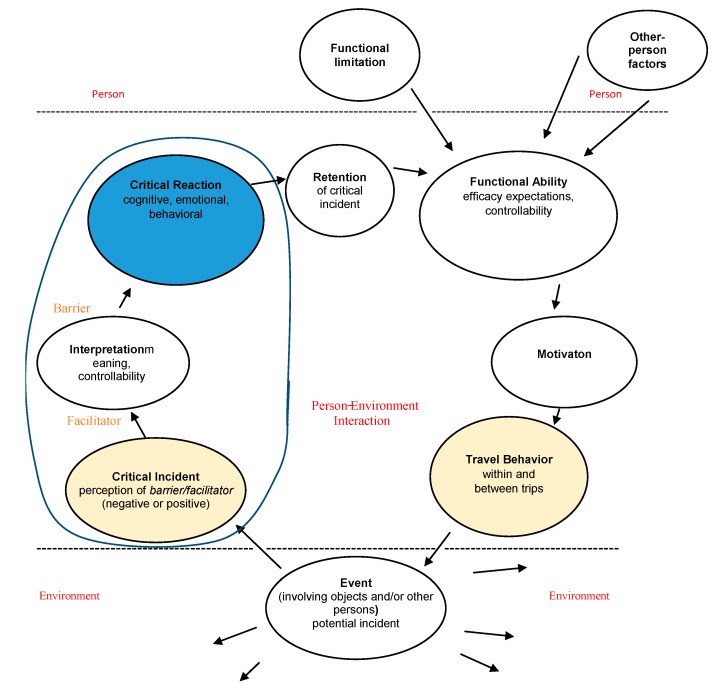
Conceptual cognitive and behavioral framework of person–environment interaction, illustrating the process of travel-behavior change in an individual.

A general conceptual framework of the *process* of travel-behavior change is presented in Figure 2; this framework is grounded in environmental as well as psychological factors at the individual level. This new model is based on Model 1a [14] and Model 1b in Figure 1, and it is grounded in cognitive and behavioral theories [13,15]. In the following, I will present and discuss the conceptual framework of travel-behavior change. The framework of Model 1b in Figure 1 is further developed and extended in Figure 2, which consists of *events* in the environment that are perceived as *critical incidents* (*i.e.*, barriers/facilitators), the travelers’ *interpretations* and *critical reactions*, followed by *retention* of the incident, perceived *functional ability*, *motivation* to travel, and actual *travel behavior* (see Figure 2).

*Events* (involving objects and/or other persons) are potentially perceived as critical incidents. Along the travel chain, travelers will selectively focus on various features of the situations encountered in travel environments. The travelers will therefore differ somewhat in what they perceive, because of differences in environmental properties (e.g., depending on travel mode) as well as individual features (e.g., depending on functional limitations, age, and gender). The travelers will individually and selectively attend to stimuli in the travel environment. What is perceived as a critical incident depends partly on the individual traveler. For example, a bus driver who stops far from the pavement [18] may create a situation that some perceive as a critical incident but that others may not even notice.

*Interpretation.* In the *interpretation* and cognitive processing of a situation, the travelers’ goals and values will affect the *meaning* ascribed to what is perceived [19]. For example, a delay on the way to an important appointment may be interpreted differently from a delay on the way home. Interpretation is partly based on experiences that are shared due to, for example, shared gender and culture, and is partly idiosyncratic [20]. Although sharing the same kind of functional limitation, different individuals may still interpret a situation in different ways. Consequently, the functional limitation is not sufficient for understanding travel behavior. Moreover, even if a barrier does not exist in the “actual” environment, it will hinder travel as long as it is present in the person’s mind [20]. Perceived control of one’s behavior *and* of its outcome will influence the interpretation of the situation [21]. Perceived external control over an outcome (that is, low personal control) may evoke psychological distress, possibly lowering the motivation to solve the problems encountered [19,22]. Thus, regardless of what is encountered in the external environment, positive and negative incidents in traveling may be created in the imagination and therefore depend on personal (e.g., personality), situational, biological, and cultural factors, as well as on earlier experiences. Older persons report a lower sense of control than do younger adults. Beyond the age of 60 years, perceived control decreases at an accelerating rate, possibly because of retirement, deteriorating health, increasing rate of widowhood [23], as well as a contracting personal network due to the death of friends and kin.

*Critical Reactions. Cognitive* reactions to incidents are influenced by interpretation and may activate both positive and negative *emotional and behavioral reactions* [13,24] or, alternatively, the emotional reaction may be automatic and therefore precede cognition [25]. In service research, emotional reactions have been demonstrated to be linked to how a service is experienced. For example, Friman [26] demonstrated that in public transport, travelers’ moods may be affected by positive as well as negative incidents. For persons with functional limitations, events in the travel environment have been found to evoke (negative) feelings of insecurity, humiliation, stress, and inferiority, but also (positive) feelings of safety [18].

*Retention.* Both the interpretation of an incident and the reaction to it affect what is remembered. Moreover, a minor incident may be enlarged in memory and therefore affect long-term behavior or, conversely, as the memory of the incident fades, its perceived importance will diminish. Therefore, the same incident might be described differently, depending on the time passed since it occurred [27].

*Functional Ability*. Although functional limitations and other personal factors, such as age and gender, may influence functional ability, they are not the only determinants. Rosenkvist [20] demonstrated that, to maintain an ability to travel, both external and internal challenges have to be kept at a manageable level. An example of an internal challenge is lack of knowledge of what will actually happen. The ability to execute a certain behavior, *i.e.*, perceived self-efficacy, may affect behavioral decisions, and different persons assess their abilities in different ways [28]. The expectation of not being able to climb the stairs, if the elevator is out of order, may affect the travel decisions made. To overcome a barrier, the traveler therefore needs actual as well as perceived ability [14]. Moreover, succeeding with less effort would strengthen perceived ability as compared with succeeding with more effort (*cf.* [29]). Efficacy expectations of one’s ability will decline after repeated encounters with barriers too severe to overcome [15].

*Motivation.* A person’s motivations for a behavior are based on cognitive expectations of outcomes and are supported by conviction strength regarding efficacy and controllability, as well as by affective factors [24,30]. According to Bandura [31], motivation is defined as “activation to action”. The level of motivation is reflected in the choice of courses of action and in the persistence of effort. The (travel) behavior may therefore be modeled by its anticipated rather than actual consequences. However, certain functional limitations may involve difficulties performing certain behaviors even if the motivation is high. Thus, *perceived* control would affect motivation and intention, but for behavior to be affected, *factual* control would be necessary [32]. If a performed behavior does not measure up to the person’s standard, it will be disregarded as unsatisfactory. After such a “failure” or “loss” situation, associated situations are certainly more likely to be avoided and may therefore limit the behavior repertoire. Travelers must therefore actively motivate themselves to choose a certain behavior.

*Travel Behavior.* In travel behavior research, Ajzen’s *Theory of Planned Behavior (TPB)* is often applied [21,33]. According to this theory, a person’s *intentions* to perform a behavior are determined by three factors: her/his (a) *attitudes* to the behavior (because of beliefs about expected consequences); (b) *normative expectations* of others (*i.e.*, social pressure) [34]; and (c) *perceived behavioral control.* However, even though perceived behavioral control influences intentions, for the behavior to be affected, actual behavioral control is needed. Although many travel decisions are planned and consistent, behavior may be less intentional, for example, impulsive [35], and therefore more difficult to predict [32]. Alternatively, travel behavior may be directed by automatic processes [36], for example, if approach or avoidance behavior automatically follows from a biased interpretation of a situation [37]. Moreover, emotions may affect travel behavior, as when being afraid of performing a behavior may result in avoidance. Mann and Abraham [38] found that utility beliefs, such as time efficiency and reliability, could influence travel-behavior decisions through their affective impact. In service research, satisfaction has been found to be an indicator of future behavior [39]. Another indicator is the relationship to the service provider, such as trust [27] and confidence because the service provider keeps promises, is reliable, and has integrity [40]. Pullman and Gross [41] found that relationships to staff to have the most influence on loyalty behaviors toward service providers. The link between relationships and loyalty is explained through the positive emotion elicited. Rosenkvist *et al.* [20] found that trust in other people in the local neighborhood is important for moving around and that the presence of friends (or other trusted individuals) is experienced as supportive in public-transport traveling. A final example is habits, which have proven useful in predicting behavior. Habits may be understood in terms of both automatic and intentional behavior [42]. Forward [43] demonstrated that past behavior is important in predicting intentions regarding car-driving violations. In a stable context, past behavior has proven to predict future behavior better than intentions do. In an unstable context and for infrequent behaviors, however, intentions predict future behavior better than past behavior does [36]. Consistency of behavior is therefore situation specific, and as the features of a situation change, different behaviors are created [24].

In sum, apart from environmental and socioeconomic factors, *travel behavior change* is dependent on psychological factors, such as attitudes, perceived control, habits, and past behavior, as well as on intentions and emotional reactions. Interpersonal factors are also expected to affect travel behavior, such as the traveler’s normative expectations of others and relationships with the service providers in terms of trust and confidence. Moreover, even if the observed behaviors are similar, motivations and intentions may vary. To conclude, the traveler’s cognitive, emotional, and behavioral processes must be taken into account to understand why an encountered incident affects a travel behavior.

### 3.2. Barriers and Facilitators in Public Transport Traveling

Several *quality attributes*, for example, reliability and frequency of service, fare prices, and speed, have been proposed that might affect the user satisfaction of “mainstream” public transport passengers [44]. Other quality attributes relate to treatment by employees, simplicity of information, safety, comfort, design, cleanliness, and not having to change vehicles [45,46,47,48,49]. Parasuraman, Zeithaml, and Berry [50] proposed five generic service dimensions, *i.e.*, reliability, assurance, tangibles, empathy, and responsiveness, which are appropriate for service research in general and not specific to travel environments [51]. Except for *tangibles*, which were not identified by the authors as a separate dimension, their remaining four dimensions were similar to those subsequently identified in a review by Edvardsson [52]. Notably, dimensions identified in other kinds of service environments might not be applicable to public transport (see further [44]). There are also variations within public-transport services [47]. Depending on the purpose of the trip, Anable and Gatersleben [53] demonstrated that the same travel mode may be perceived differently, for example, emotional factors are more important in leisure journeys than work journeys.

## 4. Method

In a previous study by Sundling *et al.* [54], interview data on critical incidents were collected to explore the barriers and facilitators older travelers encounter in public transport. That an incident is deemed critical implies that the incident has generated travel behavior change. Critical incident technique (CIT) was used (for the CIT method, see [55]), making it possible to identify critical events in the travel chain, that is, events that travelers find important without making *a priori* definitions [56]. The present research explores the link between such critical incidents and individuals’ travel behavior, expressed as cognitive, emotional, and behavioral critical reactions to the incidents, the focus being on *why* travel behavior has been affected. The travelers’ critical reactions to incidents would help explain the psychological process (Figure 2) by which the incidents are linked to future travel behavior.

### 4.1. Participants

The critical incidents were provided by 29 older participants aged 65–91 years (arithmetic mean, 75 years; year of birth, 1921–1947), 23 women and 6 men, all from the County of Stockholm, Sweden. All interviewees had some kind of functional limitation and/or degree of reduced functional ability (self-rated); most of them had more than one functional limitation (Table 1). Participants were screened using a questionnaire on age, gender, functional limitations, and travel frequency. Inclusion criteria for incidents were that the interviewee should have encountered an event that affected a trip made by public transport that was: (a) at least partly by train (all rail-bound modes), (b) taken during the last two years, and (c) within Sweden. The whole journey was included, from the planning stage to the arrival at the intended final destination. The interviewees were allowed to report an unlimited number of incidents from an unlimited number of trips.

**Table 1:** Functional limitations of the 29 participants, 65–91 years old.

Functional limitation (FL)	Frequency of FL
Restricted mobility	16
Vision impairment	13
Cardiovascular disease	10
Hearing impairment	9
Chronic pain	7
Diabetes	4
Asthma, allergy, and hypersensitivity	4
Attention, memory, and concentration disability	4
Neurological disorder	4
Chest disease	3
Mental ill-health	2
Travel sickness	2
Reading, writing, or speech disability	1
Rheumatic disease	1
Sum of FL	80
No FL	1

### 4.2. Procedure

The study was conducted in accordance with the Declaration of Helsinki. After approval from the local ethics committee (Project identification code 2011/1169-31/5), 30 post-trip semi-structured interviews were conducted. All but one, that is, 29 interviews, constitute the present dataset. One interviewee did not fulfill the inclusion criterion of having traveled by train within the last two years. One-third of the participants were recruited from an earlier questionnaire study on functional ability, barriers, and travel behavior [4]. The other interviewees were recruited through the community’s elderly care service, by advertising in a local paper, and by the snowball method [57].

Almost all the interviews took place either on the premises of Stockholm University or in the participant’s home; in one case, the interview took place at the participant’s workplace. The author served as the interviewer. Prior to each interview, the goal was stated and questions of anonymity and free consent were addressed. Incidents were defined as “occurrences that affected the interviewee’s experience of a journey.”

In the interview: (1) participants were asked to give information about an unlimited number of incidents, positive and negative, that they had experienced on trips (at least partly by any rail-bound mode) over the last two years in Sweden. Open-ended follow-up questions were asked to make the descriptions of situations detailed and vivid. The number of incidents described varied between 1 and 47 and the duration of the interview therefore varied between 15 min and 2½ hours; (2) The interviewees scaled each incident on a three-point scale according to how strongly it had affected their travel behavior. The response categories were “1: no influence,” “2: low influence,” and “3: high influence”; (3) *Antecedents* (*i.e.*, occurrences and expectations preceding the incident) were assessed; (4) The cognitive, emotional, and behavioral *reactions* to the incident were the reported explanations for the changed travel behavior.

With the informed consent of the participants, the interviews were recorded and the answers were immediately written down to ensure their descriptive validity [58]. The interview continued until the interviewees could remember no more incidents.

### 4.3. Data Analysis

Out of a total of 469 incidents collected, 77 were perceived by the interviewees as *critical incidents*, because they were scaled by the interviewees to “highly influence” travel behavior (“3” on the three-point scale), 67 in a negative way and 10 in a positive way (see Appendix for critical-incident examples). The participants’ stories are treated as reports of facts [59]. The reactions to the 77 critical incidents are referred to as “critical reactions,” because they were, like the incidents, critical for subsequent travel behavior. They constitute the present unit of analysis.

*Thematic analysis of critical reactions.* The recorded interviews were listened to repeatedly and notes were made. First, all incidents were categorized and, second, the reactions (*i.e.*, cognitive, emotional, and/or behavioral) to each incident were sorted into preliminary themes (each reaction could belong to several themes). As the analysis progressed, a more theoretical process was applied in which the critical reactions were analyzed with reference to existing literature. The critical reactions were also analyzed by a colleague and former co-author who served as an independent judge in the incident categorization process [54]. In this research paper, I report the results of the thematic analysis (see [60]) conducted to discover the process (see Figure 2) whereby critical reactions affected travel behavior according to the interviewees themselves.

## 5. Results

The critical reactions to the critical incidents encountered are regarded as mediating variables that would have a high influence on long-term travel behavior, according to the interviewees themselves. A high negative influence primarily implies that the interviewees “decreased the number of trips considerably,” “ceased to use the travel mode in question,” or “ceased to travel alone.” A high *positive* influence implies “more frequent traveling” than in the no-incident case. A large majority, 67 of the 77 critical incidents collected (or 87%), were negative, so I will focus mainly on the *critical reactions* to these incidents.

The main results are presented in the form of a classification system developed by the author and based on the critical reactions to the 67 negative incidents collected in the interviews. The critical reactions are viewed within a framework of cognitions, emotions, and (short-term) behaviors. Table 2 presents the typical cognitive, emotional, and behavioral critical reactions, according to the classification, for each of the five themes identified in the data. The negative critical cognitive, emotional, and behavioral reactions in the five themes were (Table 2): (1) firm restrictions, (2) unpredictability, (3) unfair treatment, (4) complicated trips, and (5) earlier adverse experiences. Each of the 67 (negative) critical incidents fit at least one of the five critical reaction themes. Each of these themes will be presented in the five following sections. Notably, the number of critical reactions reported was approximately equal for the themes firm restrictions, unpredictability, and complicated trips, which together account for 77% of the critical reactions reported; these mainly represent *reactions to physical barriers* in traveling.

**Table 2:** Examples of “typical” negative critical reactions (cognitions, emotions, and/or behaviors) classified in the five themes resulting from a thematic analysis.

Five Critical Reaction Themes *	Three Psychological Classes of Critical Reactions		
	Cognitive	Emotional	Behavioral
Firm restrictions (*n* = 22)	It is just not possible.	Resignation	Choose other ways of traveling
Unpredictability (*n* = 21)	What will happen? Should I travel?	Worry, insecurity, and fear	Avoid traveling Travel although afraid
Unfair treatment (*n* = 14)	It is not fair. I do not want to travel.	Anger, irritation, and humiliation	Stop traveling
Complicated trips (*n* = 19)	It is too much.	Confusion and irritation	Travel with much effort
Earlier adverse experiences (*n* = 5)	Have to be on guard.	Vigilance	Adapt travels (e.g., only daytime)

* Resulting from interpretation of interview data (two judges). Number of incidents illustrating each theme are indicated within parentheses.

### 5.1. Firm Restrictions

Several interviewees reported that they had encountered critical incidents that they interpreted as “just impossible to overcome,” that is, firm restrictions. Except for worry during the travel situation, the interviewees described few emotional reactions to firm restrictions. Steps too high to climb or luggage shelves too high to reach generated critical reactions such as “being excluded” from certain trips. For some interviewees, an alternative travel behavior was to use the Special Transport Service, for example, when traveling with both a walker and a bag. The interviewees wished for more staff to help. Firm restrictions perceived by travelers with balance problems were underground station entrances that lacked elevators or long, fast-moving escalators. When encountering these barriers, the travelers’ critical reactions were that they had been “cut off from traveling altogether” or “forced to choose alternative ways of traveling.” In an underground station, one interviewee found herself trapped halfway down the staircase. Because of the lack of handrails, she was unable to get up or down. She requested help from a fellow passenger who happened to be present and took a taxi home instead of the underground. In the long term, she changed her travel behavior by avoiding certain trips, because of the critical reactions of dizziness and the fear of falling in the travel situation. An interviewee with balance problems at stations with long escalators would travel only in company because of vertigo. If she traveled alone, she would make a detour to another underground station with shorter escalators. Other interviewees ceased to use the underground altogether, because of the fear of using escalators. For some of the interviewees, “too expensive tickets” for long-distance trains were perceived as a firm restriction that made interviewees cease traveling by long-distance train. At the time of booking, it may be important to get information on the kind of carriage to be used, for example, to avoid cramped carriages that, in the case of joint problems, might produce aching knees. The impossibility of overcoming such barriers was typically more or less taken as fact and other options had to be chosen. In the long term, a firm restriction would evoke the emotional reaction of resignation. The long-term behaviors for dealing with such barriers were either to find other ways of traveling (e.g., taxi instead of bus) or not to make the trip at all. For some interviewees, traveling in company had become the only long-term solution, resulting in dependency.

### 5.2. Unpredictability

For many travelers, unpredictable events were a source of worry and insecurity, and such events would leave the interviewees feeling helpless. A long-term consequence of this critical reaction might be apprehensive expectations before each trip: “It’s an adventure every time, it’s because of my balance. I become completely perplexed if the elevator is out of order and if there are no stairs. Then I suppose I would have to go home. I do try to calm down before a trip. I leave home earlier even though I get palpitations. Before Christmas I thought ‘I will never travel again,’ but then I forced myself, thinking that I cannot be stuck here” (Interviewee A, woman, seven functional limitations, reduced functional ability).

Upon arrival at stations, several interviewees had found the elevator out of order. An immediate critical reaction was worry, while a longer-term reaction was apprehensive expectations, and one interviewee found it difficult to sleep the night before a trip. Another interviewee got lost because she could not use the usual exit; her reaction was to “feel stupid” and stressed. Her travel behavior was affected such that she initially avoided traveling. On the train, some interviewees could not relax because they were unable to keep an eye on their luggage, which they would have preferred to have near at hand.

The interviewees sometimes did not know beforehand whether a bus driver would stop near the pavement or whether the kneeling function (which lowers the bus toward the curb) would be used at the bus stop. This would induce critical reactions of fear because they were afraid of falling, especially if the interviewees had to lift their walkers off the bus. One interviewee had fallen when the bus started moving before she was seated, and several other interviewees reported being afraid of falling in similar situations. This would evoke critical reactions of anxiety before and during the trip: “I thought I would fall. It happens so easily if you’re not young like my grandchildren, who can jump from the balcony without anything happening. The bus drivers are stressed and don’t have time. Most often, they are sympathetic, but when there are lots of passengers, there might not be enough time for them to think of it” (Interviewee B, woman, one functional limitation, somewhat reduced functional ability).

With regard to travel behavior, unpredictability may create a dilemma: whether to travel and risk encountering the barrier, or choose safety and avoid traveling. For some interviewees, a resulting travel behavior was to choose the Special Transport Service, although they could have traveled by bus if the driving had been more careful and predictable. Some interviewees stopped traveling altogether by public transport and were instead driven by car by friends and family. Some continued traveling by public transport but only in company. Others made only minor travel behavior changes such as talking to the driver instead of just pushing the stop button before getting off the bus.

Lack of information induced critical reactions of irritation and worry, for example, during delays or when managing unexpected changes in travel mode if carrying luggage. A short-term behavioral consequence for one interviewee was getting on the wrong bus. A long-term travel-behavior consequence was to travel by bus instead of train, unless one could travel by train the whole distance. During a delay, another interviewee experienced critical reactions of worry and discomfort, not because of the delay itself, but because of being unable to inform her daughter, who was about to pick her up at the destination station. These stressful critical reactions, in turn, reminded her of her heart condition and that she should avoid stress: What would happen if she became ill and needed a doctor? After this event, a long-term travel behavior was to cease travelling by long-distance train.

### 5.3. Unfair Treatment

Some interviewees developed strained relationships with transport providers because they felt that the transport system had treated them disrespectfully or unfairly. Various computerized solutions were perceived as barriers by the interviewees because they felt excluded from traveling on the same terms as others. Some interviewees did not have a computer or had difficulties using one, while others felt forced to use a computer. Some interviewees had stopped traveling by long-distance train, partly for this reason. The interpretation of “being treated unequally” followed, for example, the worry of being left alone with an impossible-to-figure-out ticket machine or having to order the ticket over the Internet to get the best price.

Some interviewees attributed the barriers partly to themselves: they felt “stupid,” thinking that they “should have been able to learn.” Importantly, personal assistance was regarded as a more flexible and predictable solution than the many specialized solutions involving electronic devices. In particular, interviewees asked for staff to help with ticket purchases to be made on the Internet, at least at larger stations. That way, they saw an opportunity to learn more about the electronic system and become more independent. One participant experienced first-class double-decker trains as similar to “cattle trucks,” because the carriages were cramped and unstable; the resulting travel behavior was that this participant would “never get onboard such a train again.” Notably, the interpretation of a situation as “unfair” evoked emotional reactions of irritation, anger, and humiliation. Ticket prices that fluctuated depending on departure time were conceived as deceptive, particularly if the ticket was booked close to departure time and, therefore, had increased in price. Long queues, faulty booking information, or windy, unprotected platforms were other critical incidents experienced as disrespectful treatment by the transport system. A resulting travel behavior was to choose the bus instead of the long-distance train.

Although these critical incidents were attributed to the (external) transport system, some travelers partly blamed themselves (*i.e.*, internal attribution) for their reactions, for example, not being able to learn how to overcome the barriers. Perceived external control (and thus low internal control) possibly impoverished the relationship with the transport provider, which was interpreted as a counterparty not interested in passenger needs. One interviewee even described this relationship as “having an enemy” and, consequently, avoided any further train travel. Some interviewees also felt insulted by the general acceptance of mobile-phone conversations onboard trains; they found themselves “listening almost compulsively,” though not wanting to, and found the situation to be “unbearable.”

### 5.4. Complicated Trips

Complex journeys involving changes of travel modes force the traveler to be alert during throughout the journey [61]. Encountering many barriers during a trip might have a cumulative effect [16,20,59], making the traveler reach a threshold beyond which it is no longer worth the effort to travel, even though each individual incident could be regarded as minor. Travel motivation will therefore decline, at least if other alternatives exist [17]. Previous incidents may serve as antecedents for latter incidents, affecting the perception of the latter. One example is the system of fluctuating ticket prices that some interviewees found difficult to grasp. Their resulting short-term travel behavior was to put an increased (and perceived unnecessary) amount of time and effort into finding the best-priced tickets. A long-term travel behavior was to travel by bus instead of train.

Encountering several incidents on the same trip could be perceived as “almost traumatic”. One interviewee missed the train because he had had to struggle up the stairs when both the escalator and elevator were out of order. He had experienced the same combination of incidents several times. The resulting travel behavior was to plan his trip accordingly, to allow extra time at the station.

Traveling connections were regarded as complicated by difficulties transporting luggage between travel modes, getting on and off buses with a walker, and finding the right platform at the railway station in time. This made some of the interviewees change their travel behavior, choosing the bus to travel the whole distance instead of bus and train or, if possible, using the Special Transport Service. Apart from the cognitive and physical effort, changing transport modes created emotional reactions of anxiety and worry in travelers who could not manage all parts of the travel chain. One interviewee, who drove his car into the city, wished he could bring his bike on the train instead (outside of rush hours). That way he could more easily reach the places in the city he wanted to visit. Cramped luggage space made trips more complicated, for example, by making interviewees use bags that are easy to stuff into cramped spaces but heavier and more uncomfortable to carry for travelers with balance problems.

In many cases of complicated trips, the interviewees had adapted their travel behavior, for example, by using other travel modes. Some interviewees still traveled in the same way, but less frequently than they would have if it had been easier. Encountering one barrier after another often induced irritation. The interviewees also reacted with confusion and stress when there was too much to deal with. If computerized solutions were regarded as complicated, the interviewees often perceived this as low internal control over the ticket purchase. They referred, as in the following case, to similar normative beliefs of others, but also put part of the blame on themselves. This resulted in low perceived functional ability and a long-term avoidance of travel: “They (*i.e.*, the operators) ignore the fact that not everybody has a computer … I have been talking to people who say ‘Buy the ticket on the computer’—no, I can’t do that! … It is too much trouble with the computer … I feel stupid … It takes probably one hour to book a ticket. I don’t think I have ever done that myself. I find the whole situation unpleasant” (Interviewee C, woman, 2 different functional limitations, somewhat reduced functional ability).

### 5.5. Earlier Adverse Experiences

Memories of earlier adverse experiences in a travel-associated situation sometimes affected travel behavior in a negative way. Such experiences were reported by a small number of interviewed women: “I would never travel by the underground after 8 p.m. I was assaulted in the underground. I watch who is entering. You are vulnerable and exposed if something happens, and nobody can see what happens. I would never travel alone. Help is far away. In the large power failure in the 80s, I happened to be stuck. I had to remain in the underground for a long time. The lady beside me panicked, and I had to help her. Then we had to walk through the whole train and there was only emergency lighting on the platform. Such thoughts go through my mind” (Interviewee D, woman, 1 functional limitation, somewhat reduced ability).

Experiences, sometimes several years old, of power failures, hassles, being harassed, or accidents in the underground had all created critical reactions of worry, vigilance, or unease. Because of these earlier experiences, events on recent trips were perceived as negative incidents that in turn affected travel behavior in various ways: interviewees would not travel alone or would choose a carriage with other women or older couples (avoiding carriages with men only), and some avoided late-evening travel altogether. The interviewees regarded themselves as fragile, compared with when they were younger: “Nowadays, I can’t run away from anyone. I have a cardiac dysrhythmia so I wouldn’t dare run … I have to be careful” (Interviewee E, woman, 2 different functional limitations, somewhat reduced functional ability).

Because of the risk of accidents, interviewees were also watchful on platforms, especially if these were crowded. This is in line with Risser, Iwarsson, and Ståhl’s [18] finding that persons with post-stroke cognitive functional limitations perceived a risk of being pushed and of losing balance.

Having once been stuck in an elevator affected the travel behavior of one interviewee. Although she had tried to tell herself “Now, you are being ridiculous, just go ahead,” she always ended up *not* entering the elevator in the underground station, unless she had someone accompanying her. This was because, in that situation, her cognitive reaction was to be “unsure of how long it would take to get help” if she became stuck. She was fully aware that she was overemphasizing a low-probability outcome and letting emotion drive her into irrational behavior.

Because of earlier experiences, the interviewees generally wished for more guards on platforms and in trains to counteract their reluctance to travel. They welcomed camera surveillance and the presence of staff overseeing activities.

### 5.6. Positive Critical Reactions

Out of the 77 critical incidents that greatly influenced travel behavior, only 10 were positive. Just as firm restrictions, such as expensive tickets, were reasons why some older persons did not travel, other older persons had the opposite experience, for example, inexpensive tickets being regarded as opportunities and constituting reasons for traveling more often. Travel costs were more important than, for example, travel time, a result in line with Su and Bell [62]. The interviewees differed greatly in their observation and interpretation of whether or not a situation was experienced as “unfair treatment.” What was experienced as unfair treatment or complicated travel conditions by some were regarded as advantages by others; for example, fluctuating prices, which some regarded negatively, were appreciated by others because they enabled them to buy inexpensive tickets if booked well in advance or at the last minute. Likewise, credit card payment on the phone made it easier to travel, as did frequent departures in the underground, which was moreover experienced as a security factor. One example of *earlier positive experience* or *consistency* making travel possible is when a traveler knows from experience that staff will be available to help at a particular station.

Some interviewees preferred the mostly aboveground commuter trains to the underground, because they could look out the windows at scenery and would feel less trapped inside the train if something should happen. Punctuality and low prices were also appreciated by the interviewees and therefore created positive critical reactions.

## 6. Discussion

The primary objective of this research was to study the process of travel-behavior change within a cognitive and behavioral framework of person–environment interaction. By examining the interviewees’ critical cognitive, emotional, and behavioral reactions to critical incidents, it was possible to identify why certain incidents resulted in changed travel behavior according to the travelers themselves. It should be noted that though the present sample of older travelers is relatively small, it is still heterogeneous. The themes identified are partly in line with categories identified by Friman [26] (*i.e.*, unpredictability and unfair treatment) as regards negative critical incidents reported by “mainstream” travelers on public transport. However, the vulnerable travelers studied here also encounter other difficulties than those affecting mainstream travelers, that is, difficulties perceived as “firm restrictions” that cannot be overcome, as also found by Jensen *et al.* [16] in their accessibility research on bus travel among persons with functional limitations.

### 6.1. Control

The present data suggest that the feeling of being in control is central. Encountering unpredictable events in the travel environment was found to result in critical reactions of insecurity, because of uncertainty as to whether the trip would be manageable. Not knowing what to expect creates critical reactions of stress, especially if the purpose of the trip is important and, in addition, if the risks involve the person’s own health, such as the risk of falling. For example, some of the “older” old interviewees (*i.e.*, 75 years and older) and those who used a walker were afraid of falling when getting off a bus. If an incident is interpreted as exceeding the perceived functional ability, the outcome in terms of travel behavior may be to avoid similar situations, because we tend to choose situations we believe we can handle. A more careful and consistent bus driving style (e.g., always stopping near the pavement and using the kneeling function) would increase the traveler’s sense of being in control and perceived functional ability in that travel situation. Similar driving style problems have been reported in earlier research and driver training has been suggested as a response [17,18,20]. Likewise, not trusting escalators and elevators, and having no option if they are broken, means that vulnerable travelers cannot be certain of completing the trip and reaching the destination. Notably, interviewees wanted repairs of such breakdowns to be swift. These results indicate that even if there is “formal” access (e.g., elevators installed), “actual” access will still be perceived as low as long as it cannot be depended on. This is in line with earlier research arguing that confidence is essential for customers (here, travelers) in their interactions with service providers [40]. Not only may events observable by others and obviously seriously adverse, such as harassment, result in a critical reaction of vigilance, but also seemingly smaller unpredictable events occurring in the travel environment (e.g., due to driving style) may be central because of the older travelers’ exposed situation. Moreover, a seemingly small incident may have severe consequences if it is enlarged in the traveler’s interpretation and memory [63], and therefore affect long-term behavior, although a general bias can be expected toward more recent incidents that are easier to recall. Because of the time period of two years used in this research, the reaction to an incident might have been described in different ways, depending on the time passed since it occurred [27]; conducting the interviews at another point in time might have given partly different results.

In the long term, insecurity and worry before a trip may initiate a downward spiral in travel behavior in which travelers might seek reassurance from family or friends, in turn creating even more dependence. Having to adapt one’s traveling to other persons’ habits may decrease travel frequency and increase social exclusion, if traveling is restricted to the opportunities when company is available. Some interviewees had indeed stopped traveling by public transport and had consequently become dependent on other persons. Gradually lower efficacy expectations of one’s functional ability may follow (because of fewer opportunities to learn) and possibly a more adverse self-perception; for example, some interviewees put part of the blame on themselves for not managing to travel.

### 6.2. Emotion

Emotional critical reactions were central to travel behavior in many interviewees. For some interviewees, the actual critical reaction (e.g., of stress and worry) became in itself a barrier to travel, for example, if the person had a heart and/or chest condition. Notably, not only obviously severe events such as hassles or accidents but also even seemingly mildly adverse events may evoke equal fear in the individual. This could apply if the event was not even noticed by other persons present. For instance, a person who has repeatedly experienced fear of falling when getting off the bus, or “feeling stupid” because of difficulties with ticket purchase or non-functioning elevators, may develop a vigilance for as well as lowered perceived functional ability in similar situations. This humiliating interpretation of inferiority *vis-à-vis* the transport system and of being “stupid in traveling” has also been found in previous research into persons with functional limitations [18]. If an individual wants to be more mobile than he or she perceives is possible, dissatisfaction will follow [20]. Although the interviewees’ emotional and cognitive critical reactions were usually in line with their “choice” of travel behavior, in some cases, the emotional reaction “takes over” and affects travel behavior against the traveler’s intentions. For example, because of earlier adverse experiences, fear of being left alone in an elevator resulted, for one interviewee, in impulsive avoidance of that behavior, despite the initial intention to use the elevator and her long-term wish to do so. Likewise, the humiliation of entering what is perceived to be a “cattle truck”-like train, deemed “unfair treatment,” caused another interviewee to avoid traveling by train, although he would have preferred this travel mode under other circumstances. His humiliation is an example of a more planned avoidance behavior than the former elevator example. Such unpleasant emotions may result in avoidance behavior, whether impulsive or planned, although this behavior might not be “rational.” In the latter case, however, it was not cognitive critical reactions to a barrier exceeding functional ability that restricted the interviewee’s travel behavior. If the traveler felt “fooled” or forced into an unwanted situation (“unfair treatment”), he did *not want* to travel under those circumstances even though they *could* travel. In either case, avoidance may result in a more limited behavior repertoire. Therefore, not only the incidents per se, but also the reactions to them, including emotions and attitudes, must be considered by transport providers in order to better understand older travelers’ behaviors and needs. In line with the present research, Rosenkvist *et al.* [20] found that even imagined barriers could also result in difficulties using public transport. Pullman and Gross [41] found relational aspects, explained by the emotion elicited, to be the strongest promoters of loyalty behaviors toward service providers. Moreover, emotional factors have been demonstrated to be more important in leisure journeys than in work journeys [53]. As barriers are not always obvious to others, transport providers should become informed of travelers’, and potential travelers’, needs, as suggested by Rosenkvist [20].

### 6.3. Staff

The data suggest that absence of staff is central to travel behavior in a number of ways. Available staff is perceived as a general security factor, that is, “to have someone to ask in case something unexpected happens.” Moreover, personal service is viewed as far more flexible than electronic solutions (e.g., regarding ticketing), and some interviewees interpret the lack of alternatives as unfair treatment, because not all travelers have the same opportunities. According to earlier research, staff availability during trips is experienced as more important for older than for younger persons; especially for older persons, technical solutions cannot generally replace personal service (*cf.* [6]). As also demonstrated by Rosenkvist *et al.* [20], staff may help bridge the gap between the traveler’s decreasing ability and the increasing perceived environmental demands. As well as staff, the presence of other persons whom the travelers could trust was experienced as supportive.

### 6.4. Heterogeneity

There was considerable heterogeneity in the group of older persons. In this research, many of the “older old” (75+ years old) interviewees were experiencing increasing frailty, often using aids such as walkers, and had encountered certain critical incidents and reacted to them accordingly, while many of the “younger old” interviewees had encountered other kinds of incidents. Moreover, only the women (23 out of 29 in this sample) identified critical incidents based on earlier “frightening” events, sometimes experienced many years previously, that still made them react with vigilance and insecurity in similar situations. It was the women who used walkers, and therefore experienced great difficulties getting on and off buses, and were moreover the oldest interviewees. Even nationally, it is more common for women than men to use walkers [64]. Notably, in many countries (e.g., Sweden), most of the older old are currently, and will in the future, be women, although the life-expectancy gap between men and women is narrowing in some parts of the world [65]. Moreover, what is perceived as a negative incident by some travelers can be perceived as positive by others; for example, fluctuating ticket prices could be perceived as either a barrier or a facilitator. In some cases, the use of inclusive design (e.g., doors that open automatically or zero-step entrances) meets the needs of older users (*cf.* [16]). Assessing more general underlying human needs, such as a feeling of being in control and being treated respectfully, in relation to the travel environment would be one step toward pinpointing fields to be highlighted in order to better understand the reasons for people’s travel choices.

## 7. Conclusions

A predictable travel environment in which the vulnerable traveler perceives a high level of behavioral control was found to be essential for the choice of travel behavior. One way of achieving better predictability would be to increase the access to personal service throughout the travel chain, for example, by providing alternatives to electronic solutions, more visible staff, or “help telephones” at strategic places. The present research suggests that travel behavior decisions are not based solely on “rational” decisions. Therefore, travelers’ emotional experiences of the travel environment, “irrational” reasons for decision making, as well as relational factors should all be considered when designing services. Not only the travelers’ actual ability but also their perceived ability affects their travel choices. Knowing, before a trip, that staff will be present and visible would enhance the motivation to travel for many of the interviewees, helping maintain flexible travel behavior. Extended personal service along the travel chain would increase the traveler’s sense of control, helping reduce critical reactions of worry, decrease travel complexity, and therefore also decrease the effort needed. Making public transport more attractive, for “mainstream” users as well, is important and will promote increased sustainability. Such improvement might divert travelers from excessive car use to travel via public transport, reducing environmental problems such as congestion and air pollution.

## Appendix

**Table A1:** Examples of the 77 critical incidents collected and categorized in the travel-chain and travel-environment dimensions, arranged in the sequence of the travel chain during a trip.

Number of Critical Incidents (Negative/Positive) in Travel Chain Categories	Examples of Incidents, Negative (–) and Positive (+)
1. Ticketing (sum 17): Pricing (6, 3)	Prices vary depending on time of booking. Feeling deceived. (–) Inexpensive tickets. (+)
System flexibility (2, 1)	Was supposed to buy tickets on the Internet to get updated information, best price, to choose my seat. Not everyone has Internet. (–) Can pay with credit card on the phone. (+)
Information (4, 0)	Did not know that the train would be replaced by bus for part of the distance. If I had known, I would not have booked. (–)
Time and connections (1, 0)	Long waiting time for booking at station. (–)
2. To and from station (sum 2): Pricing (1, 0)	Expensive parking in the daytime. Try to travel in other ways. (–)
Physical environment (1, 0)	Pavements not sanded. (–)
3. At station (sum 18): Physical environment (12, 0)	The elevator out of order. Cannot travel by underground—what if the elevator is out of order again? (–)
Information (1, 0)	Difficult to find your way, poor signposting. (–)
Staff (4, 1)	No staff to help with luggage; impossible to travel alone. (–) The ticket collector helped me all the way to the train. Then I could manage to travel. (+)
4. On and off vehicle (sum 6): Physical environment (2, 0)	Difficult to get on bus with walker and groceries; have to lift the walker. (–)
Staff (4, 0)	Bus driver does not use the kneeling function although I have a walker. (–)
5. Onboard (sum 22): System flexibility (1, 0)	Not allowed to bring a bicycle on train, so I have to take the car into the city. (–)
Physical environment (13, 3)	The bus is lurching, making it difficult to cope with a stiff leg. (–) Nice environment on commuter trains and long-distance trains. Can look out and move around. (+)
Information (1, 0)	Train stopped. No information about the delay, the reason for it, or how long it would be. Couldn’t tell my daughter when to come and fetch me. Wondered what had happened. Stressful. (–)
Fellow passengers (2, 0)	Noisy school classes. Nobody tells them to be quiet. I got off. (–)
Staff (2, 0)	Bus driver started before I had sat down. (–)
6. More than one part of trip (sum 12):	
Physical environment (1, 0)	Train reminds me of steam locomotives from childhood; connected to bad memories. Makes me feel sick. (–)
Information (1, 0)	Misinformation. I had been told that the train manager would help me but that was wrong. (–)
Fellow passengers (3, 0)	Avoid certain underground stations late at night. Feel unsafe if there are few persons or only men on the platform. (–)
Staff (1, 0)	No staff to help with luggage and walkers. Cannot travel on my own. Have problems with my back and cannot lift my case. (–)
Time and connections (4, 2)	Several changes of travel modes to my destination. Therefore, I use the Special Transport Service [*i.e.*, taxi service for persons with functional limitations]. (–)
